# Navigating complex clinical decisions: kidney transplantation following abdominal aorto-aortic bypass in infantile Takayasu arteritis

**DOI:** 10.1007/s00467-025-06932-w

**Published:** 2025-09-11

**Authors:** Moran Plonsky Toder, Rami Tibi, Ran Steinberg, Tony Karram, Aharon Hoffman, Dawn Coleman, Irina Libinson-Zebegret, Renata Yakubov, Israel Eisenstein, Daniella Magen, Shirley Pollack

**Affiliations:** 1 Rappaport Faculty of Medicine, Technion Israel Institute of Technology, Haifa, Israel; 2 Ruth Rappaport Children’s Hospital, Rambam Health Care Campus, Pediatric Nephrology Institute, Haifa, Israel; 3 Department of Pediatric Surgery, Ruth Rappaport Children’s Hospital, Rambam Health Care Campus, Haifa, Israel; 4 Department of Vascular Surgery, Rambam Health Care Campus, Haifa, Israel; 5 Department of Surgery, Section of Vascular Surgery, University of Michigan, Ann Arbor, MI USA; 6 Department of Surgery, Division of Vascular Surgery, Duke University, Durham, NC USA

**Keywords:** Infantile Takayasu arteritis, Aorto-aortic bypass, Pediatric kidney transplantation

## Abstract

**Background:**

Takayasu arteritis (TAK) is a granulomatous large-vessel vasculitis typically affecting young adult females. Pediatric cases are rare, and infantile onset is exceptional. Management relies on immunosuppression, with surgery reserved for severe complications.

**Case report:**

We describe a now 5.5-year-old boy diagnosed with TAK at six months of age, presenting with hypertensive encephalopathy and kidney dysfunction. Despite treatment with corticosteroids and anti-TNFα, his kidney function deteriorated, leading to kidney failure and dialysis. At nearly three years of age, he underwent abdominal aorto-aortic bypass and bilateral nephrectomy due to progressive vascular narrowing and refractory hypertension. At age four, he successfully received a deceased-donor kidney transplant. Eighteen months post-transplant, he maintains excellent graft function and shows no signs of TAK recurrence.

**Clinical significance:**

This case underscores the complexity of diagnosing and managing infantile TAK with multiorgan involvement. To our knowledge, he is among the youngest reported TAK patients to undergo successful kidney transplantation following major vascular surgery. His course demonstrates the potential for long-term remission and safe transplantation under standard immunosuppression, without continued anti-TNFα therapy. The literature is sparse regarding kidney failure and transplantation in TAK, particularly in infants.

**Key management points:**

This case highlights key management dilemmas in infantile TAK, including clinical diagnosis, timing of surgery and transplantation, choice of immunosuppression, and long-term monitoring. It emphasizes the importance of a multidisciplinary approach and the need for collaborative research to address knowledge gaps in this rare but complex condition.

**Graphical abstract:**

**Figure:**
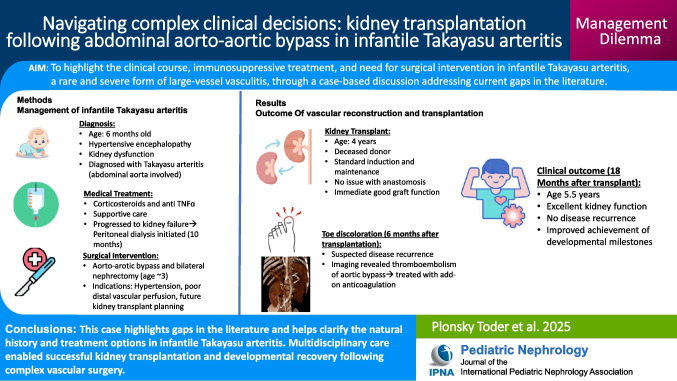
A higher resolution version of the Graphical abstract is available as Supplementary information

**Supplementary Information:**

The online version contains supplementary material available at 10.1007/s00467-025-06932-w.

## Introduction

Takayasu arteritis (TAK) is a rare large-vessel granulomatous vasculitis of unknown etiology, primarily affecting the aorta and its branches [1–3]. Although the most common large-vessel vasculitis in children, its prevalence in infants is extremely low [1].

Infantile TAK, defined as onset before 5 years, has been reported in ~ 30 cases worldwide [4–6]. Hypertension and constitutional symptoms are common, often involving the carotid, aorta, and renal arteries [4, 6, 7]. Diagnosis is challenging, relying on clinical, imaging, and laboratory findings [2, 8].

Management includes glucocorticoids and immunosuppressive agents (mycophenolate mofetil (MMF), anti-TNFα, anti-IL-6, methotrexate (MTX), and cyclophosphamide) [4–6]. Surgical intervention is reserved for complex cases [8, 9]. Most infants require multiple immunosuppressive therapies to reach remission [6].

Hypertension affects up to 92% of cases [3, 4, 6, 7], and CKD occurs in ~ 31% of pediatric TAK cases [3], though kidney failure requiring kidney replacement is rare [10]. No transplants in patients with infantile TAK have been reported [11–14]. Moreover, we were unable to find any data on TAK recurrence following kidney transplantation in any age group.

To our knowledge, this is the first documented case of severe infantile TAK leading to kidney failure and successful transplantation.

## Case outline

### Presentation

A previously healthy 6-month-old boy presented with hypertensive encephalopathy, preceded by a 3-day history of vomiting, irritability alternating with lethargy, and poor feeding, without fever. His history was unremarkable, except for G6PD deficiency. On initial evaluation, he appeared ill and demonstrated alternating irritability and lethargy but showed no signs of meningeal irritation. Vital signs revealed sinus tachycardia (heart rate 190 bpm), normal temperature, and oxygen saturation but severe hypertension (150/120 mmHg in all limbs initially), with a subsequent drop in lower limb blood pressure. On initial physical examination, the only notable finding was irritability; however, further assessment revealed diminished femoral pulses. The remainder of the physical examination was unremarkable. Initial imaging, including chest and abdominal X-rays and a head CT scan, was normal.

The complete blood count showed leukocytosis (16,000/µL with 10% PMNs), hemoconcentration (hemoglobin 15.3 g/dL), and mildly elevated platelet count (482,000/µL). Blood chemistry revealed impaired kidney function (creatinine 1.0 mg/dL, blood urea nitrogen 20 mg/dL), mild hyponatremia (sodium 129 mEq/L), normal potassium (3.8 mEq/L), and hyperglycemia (glucose 220 mg/dL). CRP was also elevated (~ 2.0 mg/dL; normal < 0.5 mg/dL). IGG was low 94.6 mg/dL (normal range 680–1560). Blood gas analysis demonstrated metabolic acidosis with low serum bicarbonate (13.5 mEq/L), elevated lactate (3.0 mmol/L), and a high anion gap of 15. Urinalysis revealed significant proteinuria (600 mg/dL), glycosuria (in the context of hyperglycemia), and mild microhematuria.

Further evaluation revealed elevated aldosterone (renin unavailable), with normal cortisol, thyroid function, calcium, and urine metanephrines. ESR was unavailable; IL-6 taken as part of the rheumatologic workup was also elevated (34.5 pg/ml, normal range < 17.4).

### Imaging and diagnosis

**Fig. 1:**
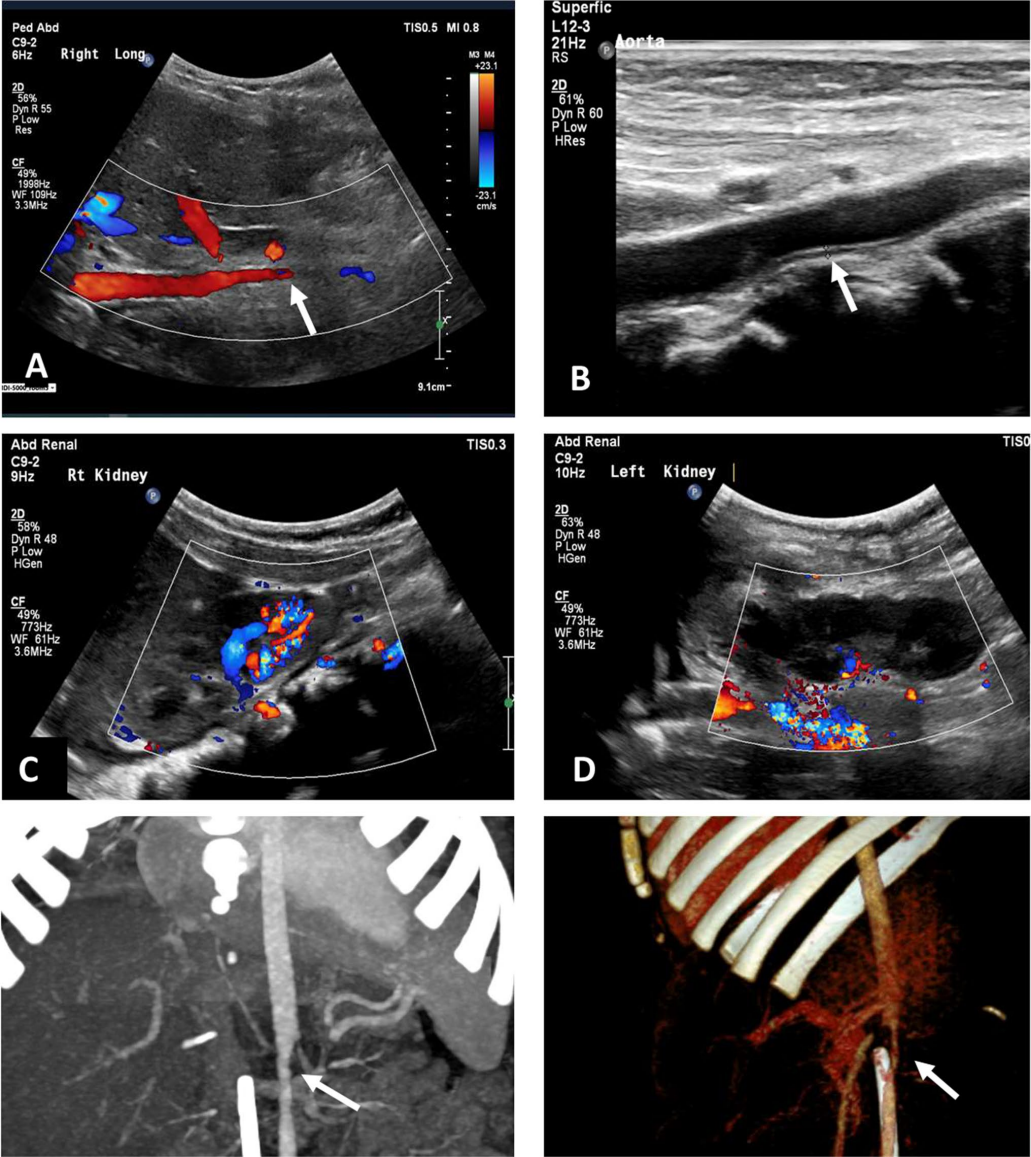
Doppler ultrasound of abdominal aorta and native kidneys at presentation. **A** Color Doppler ultrasound showing significant narrowing of the abdominal aorta lumen (arrow). **B** Gray-scale ultrasound of the abdominal aorta highlighting lamination of the vessel wall (arrow), suggestive of an inflammatory process. **C** Color Doppler ultrasound of the right kidney demonstrating vascularization limited to the lower pole. **D** Color Doppler ultrasound of the left kidney showing absent vascularization throughout the entire kidney. **E** CTA abdomen, coronal view showing segmental aortic wall thickening, enhancement, and stenosis (arrow)*.* **F** 3D “snapshot vascular” lateral view with arrow highlighting the stenotic aortic segment

Abdominal Doppler ultrasound (US) revealed a narrowed aorta with wall irregularity and poor kidney perfusion (Fig. 1A–D). CT angiography (CTA) demonstrated similar findings with disease of the abdominal aorta, characterized by wall thickening and enhancement with mild luminal narrowing (< 50% stenosis) (Fig. 1E, F). Critical stenosis was noted at the origin of the superior mesenteric artery (SMA), and only the right lower renal artery appeared patent at its origin from the aorta. No abnormalities were identified in vessels above the abdominal aorta, including the neck, thoracic, and cerebral vasculature. Importantly, CTA did not reveal features characteristic of fibromuscular dysplasia, such as a “string of beads” appearance, dissections, or aneurysms.

While the initial clinical impression suggested mid-aortic syndrome, the differential diagnosis remained broad. Considerations included congenital vascular anomalies, fibromuscular dysplasia (FMD), genetic syndromes (e.g., neurofibromatosis, Williams syndrome, Blau syndrome due to NOD2 mutation), and inflammatory diseases (such as Takayasu arteritis, COVID-19–related multisystem inflammatory syndrome, and other large-vessel vasculitides).

**Fig. 2:**
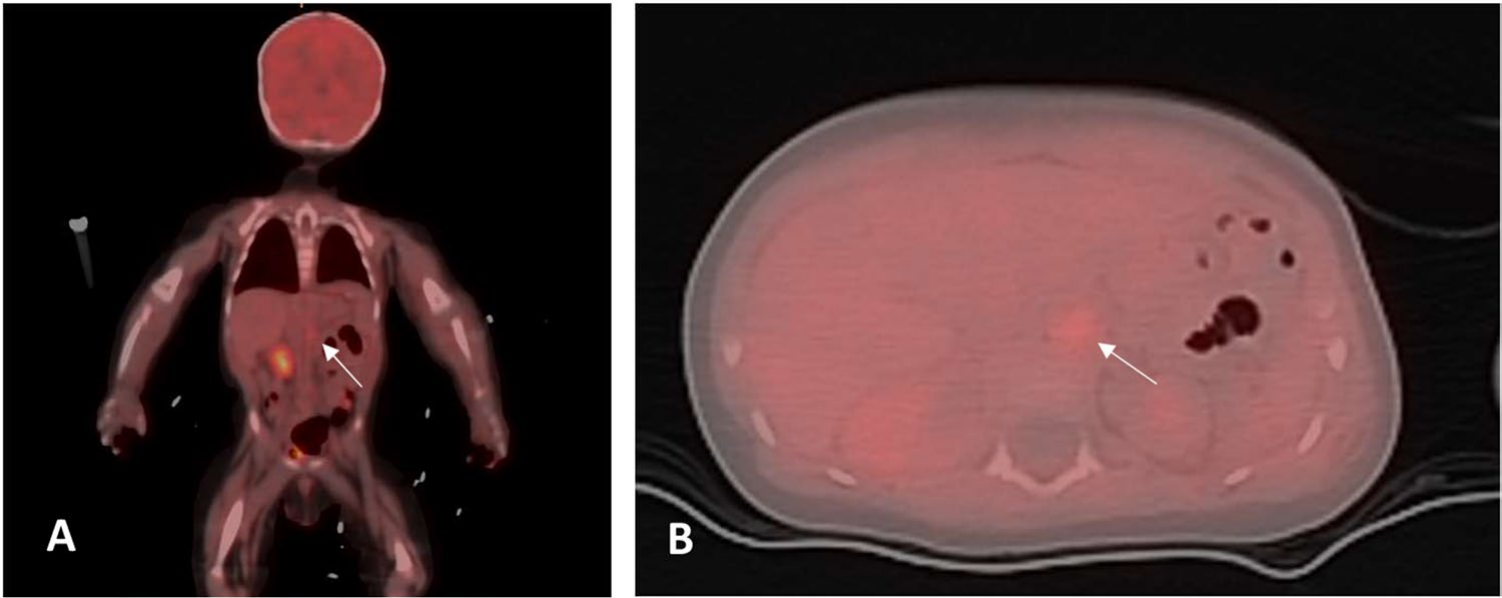
FDG-PET/CT findings at diagnosis. **A** Coronal view and **B** axial view demonstrate focal pathological FDG uptake in the abdominal aorta (white arrows), just below the origin of the superior mesenteric artery. This uptake is consistent with active inflammation and supports the diagnosis of large-vessel vasculitis, specifically Takayasu arteritis

Exome sequencing ruled out known genetic causes, and infectious etiologies, including COVID-19, were excluded. PET-CT demonstrated focal pathological uptake in the abdominal aorta—likely below the SMA (Fig. 2). Arterial fluorodeoxyglucose (FDG) uptake demonstrated an SUVmax of 2.0 in the abdominal aorta, exceeding hepatic uptake (SUVmax 1.4), consistent with active large-vessel inflammation suggestive of aortitis [15]. Additionally, there was absent cortical uptake in the upper pole of the left kidney and significantly decreased uptake in the upper pole of the right kidney, suggesting impaired perfusion. Of note, interpretation of PET-CT in children is often more challenging than in adults due to age-related differences in background uptake, suboptimal preparation—particularly difficulty avoiding glucose-containing infusions prior to imaging—and motion artifacts, all of which can compromise image accuracy [16].

Given the imaging findings consistent with large-vessel vasculitis, the absence of clear-cut features typical of fibromuscular dysplasia (FMD), and the fulfillment of both EULAR/PRINTO/PRES and ACR/EULAR classification criteria for TAK, the diagnosis of childhood-onset TAK was favored.

### Treatment and complications

Initial management included intravenous hydration and antihypertensive therapy. Blood pressure control was particularly challenging and required multiple agents from different pharmacologic classes. Initial treatment included IV hydralazine, a continuous IV labetalol infusion, and enteral nifedipine. Due to persistent severe hypertension, two separate 3-day courses of IV nitroprusside were administered, along with a week of IV phentolamine and IV nicardipine. Once blood pressure stabilized, the regimen was transitioned to oral agents.

Immunosuppressive induction therapy for suspected TAK included intravenous methylprednisolone pulses, followed by oral prednisolone, infliximab (anti-TNFα), and IVIG due to hypogammaglobulinemia.

**Fig. 3:**
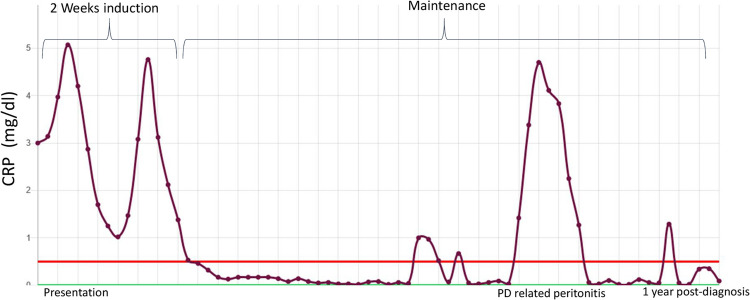
Trend of C-reactive protein (CRP) levels over time, showing marked improvement following initiation of therapy and resolution of inflammation

The initial hospitalization lasted 36 days. At discharge, the patient was prescribed a tapering steroid regimen, monthly infliximab infusions, and an intensive antihypertensive regimen consisting of six oral agents, all given at doses higher than standard recommendations: nifedipine, clonidine, labetalol, hydralazine, and amlodipine. Despite bilateral renal artery involvement, enalapril was added, as ACE inhibition was considered essential for achieving adequate blood pressure control. During follow-up, inflammatory markers normalized (Fig. 3). However, the patient required several readmissions for blood pressure management, prompting the addition of doxazosin and minoxidil to his regimen. Vascular intervention was initially deferred in favor of medical therapy to control the active inflammatory process. This decision was based on the clinical judgment that performing angioplasty during active inflammation carries increased procedural risk, including vessel fragility, a higher likelihood of arterial dissection or rupture, impaired healing, and restenosis. The angiographic team concurred that any intervention would be safer once the inflammation had subsided.

Progressive kidney failure (creatinine 3.5 mg/dL, BUN 51 mg/dL) combined with oliguria and hyperkalemia prompted initiation of peritoneal dialysis (PD) at 10 months. During dialysis, he developed symptomatic bilateral inguinal hernias, bacterial peritonitis, and global developmental delay (impaired gross motor and language skills), eating aversion requiring NG feeding and stunted growth (BMI ~ 50th percentile, height < 3rd percentile). These complications stemmed from his illness, glucocorticoids, and PD.

At 2.5 years of age, following nearly 2 years of close monitoring, he remained clinically stable with consistently normal inflammatory markers (CRP < 0.5 mg/dL) and was well-maintained on peritoneal dialysis. Kidney size gradually increased from 5.7 cm (right) and 4.3 cm (left) at presentation to 6.5 cm and 6.0 cm, respectively. However, serial ultrasounds showed increasing echogenicity and worsening corticomedullary differentiation in both kidneys. A follow-up PET-CT confirmed disease burnout.

Although serial imaging and clinical monitoring demonstrated sustained remission, severe aortic narrowing persisted. At that point, angioplasty was reevaluated but ultimately deemed technically unfeasible due to the extensive and diffuse nature of the aortic involvement. Surgical repair was therefore undertaken as the most viable option for definitive management.

To ensure long-term adequate blood supply to the gastrointestinal tract and lower extremities and to facilitate future kidney transplantation, he underwent an aorto-aortic bypass with a polytetrafluoroethylene (PTFE) graft at Mott Children’s Hospital (Michigan, USA), placed end-to-side from the supraceliac region to the terminal aorta. Prior to the surgery, he was switched from PD to hemodialysis (HD) to enable abdominal surgery. His left kidney was atrophic and removed. In an attempt to preserve residual kidney function, a right renal artery reimplantation was performed directly onto the terminal aorta.

The reimplanted right renal artery thrombosed early in the postoperative period despite appropriate anticoagulation, likely due to poor outflow. Due to refractory hypertension, a laparoscopic nephrectomy was performed 3 weeks later, prior to hospital discharge. The patient remained dependent on hemodialysis and continued treatment with infliximab, aspirin for antiplatelet therapy, and multiple antihypertensive medications.

One year later, USD and CTA confirmed widely patent aortic bypass and iliac arteries; iliac diameter of 3–4 mm was felt acceptable for transplantation. At 4 years of age, he underwent a successful deceased-donor transplant, with graft vessels anastomosed to the right common iliac artery and distal IVC. Induction included basiliximab and high-dose steroids, followed by triple immunosuppression (steroids, MMF, tacrolimus).

**Fig. 4:**
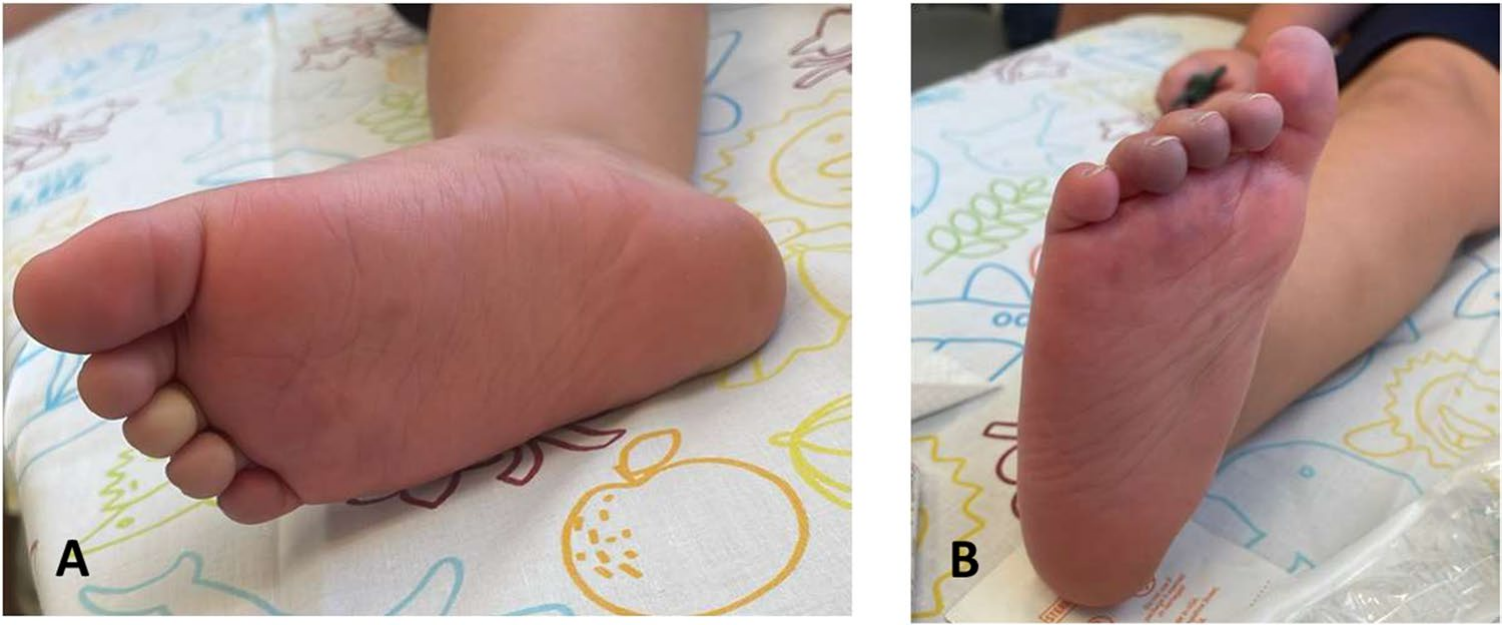
Progressive discoloration of the right foot following kidney transplantation. **A**, **B** Sequential photographs of the right foot taken several hours apart show evolving discoloration—initial blanching followed by bluish hues of the toes. Given the patient’s vascular history, this raised concern for either thromboembolism or TAK recurrence. Vascular imaging was promptly performed to differentiate between these possibilities

**Fig. 5:**
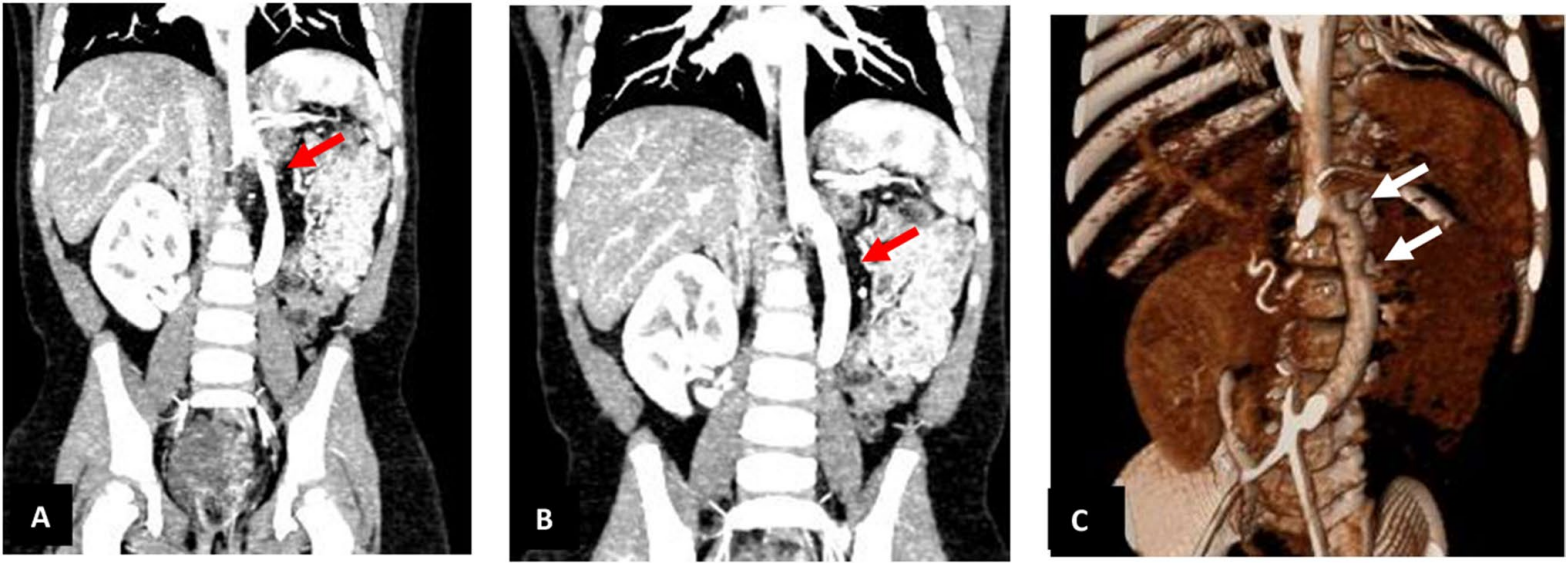
Imaging of the aorto-aortic bypass with thromboembolism. **A**, **B** Contrast-enhanced CT in coronal views showing two locations of thromboembolism along the aorto-aortic bypass (red arrows). Kidney transplant is shown in the right abdominal cavity. **C** 3D reconstruction of the CT scan illustrating the entire graft with two prominent thromboembolic sites (white arrows)

Post-transplant, early BKV viremia was managed with MMF discontinuation, leflunomide, and IVIG. Nearly 6 months post-transplant, acute toe discoloration and abdominal pain raised concern for TAK relapse (Fig. 4). However, imaging confirmed thromboembolism within the aortic graft (Fig. 5), which was successfully treated by adding anticoagulation to ongoing antiplatelet therapy.

**Fig. 6:**
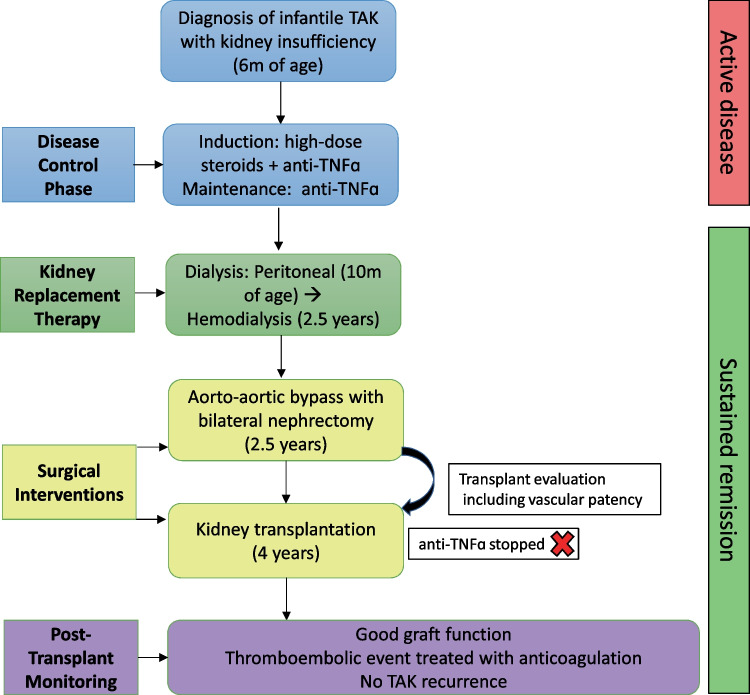
Stepwise care journey of an infant with Takayasu arteritis and kidney failure. This timeline illustrates the patient’s clinical journey from initial presentation through diagnosis, disease-modifying treatment, progression to kidney failure, vascular reconstruction, and successful kidney transplantation

At 18 months post-transplant, he is in good condition with normal graft function (creatinine 0.48 mg/dL, urine protein/creatinine 0.26 g/g, BMI > 97th percentile for age, weight 25th percentile, height < 3rd percentile) and significant developmental progress. Figure 6 illustrates the patient’s clinical journey, highlighting key diagnostic, therapeutic, and surgical milestones.

## Discussion

Infantile TAK is an exceptionally rare condition, presenting significant diagnostic and therapeutic challenges. The patient’s clinical course involved several key challenges.

### Diagnostic challenges

Mid-aortic syndrome, as previously discussed, encompasses a broad differential diagnosis. While infectious and known genetic causes can often be ruled out with relative confidence, distinguishing between FMD and TAK remains particularly challenging. In our patient, several features were inconsistent with FMD, including the absence of aneurysms, lack of the characteristic “string-of-beads” appearance on imaging, no additional vascular involvement, and no angiographic features typically associated with the condition. In contrast, elevated inflammatory markers, imaging findings consistent with large-vessel vasculitis, and increased FDG uptake on PET favored the diagnosis of TAK. This diagnosis was ultimately supported by fulfillment of the EULAR/PRINTO/PRES criteria, alongside clinical and imaging features that rendered alternative diagnoses, including FMD, less probable.

### Treatment challenges

Due to limited pediatric data, treatment followed adult TAK management [5, 9]. According to the ACR guidelines [9], induction therapy for TAK consists of high-dose steroids combined with non-glucocorticoid immunosuppressants such as methotrexate, azathioprine, or anti-TNFɑ agents. Given its relatively favorable side effect profile and promising data from infant case series [7, 8], we chose anti-TNFɑ therapy for both induction and maintenance. Steroids were gradually tapered following induction.

### Dialysis and surgical considerations

Kidney failure likely resulted from renal artery involvement and the effects of antihypertensive medications. PD, the preferred infantile modality [17], had to be transitioned to hemodialysis to facilitate vascular surgery. Vascular intervention was deliberately delayed until after inflammation had subsided and was carefully planned to ensure long-term adequate blood supply to the gastrointestinal tract and lower extremities while also accommodating future kidney transplantation. Although the persistent narrowing of the abdominal aorta remained asymptomatic during the PD treatment period, it was anticipated to potentially compromise perfusion of the transplanted kidney or cause ischemic complications in the GI tract or lower extremities.

To secure long-term adequate blood flow to the lower body and facilitate future kidney transplantation, an abdominal aorto-aortic bypass was performed. Although an attempted right renal auto-transplantation was unsuccessful due to thrombosis, the bypass graft ensured sustained vascular stability. The patient was subsequently maintained on long-term aspirin therapy to provide antiplatelet protection.

### Transplantation challenges

Kidney transplantation in TAK poses unique challenges, particularly with vascular anastomoses and disease recurrence. Considered options included anastomosis to the iliac vessels or the upper abdominal aorta above the graft. A key concern was the potential “steal” phenomenon, compromising blood flow to vital organs. However, reports in adults showed preserved lower extremity perfusion despite disrupted iliac arteries [14]. Standard iliac and IVC anastomoses were performed without technical difficulties or compromised blood flow. Standard anticoagulation after transplantation included heparin and lower molecular weight heparin in the first few days followed by long-term antiplatelet therapy. However, 6 months post-transplant, aortic graft thrombosis with distal limb ischemia occurred, prompting escalation to include anticoagulation alongside the ongoing antiplatelet therapy, with no recurrence. Hematological workup revealed no identifiable thrombophilic risk factors, either genetic or immunologic. This raises the question of whether routine, long-term dual therapy with anticoagulation and antiplatelet agents should be considered in similar patients following transplantation.

### Kidney transplantation in TAK: limited evidence and follow-up strategy

The literature on kidney transplantation in TAK is scarce, consisting primarily of adult case reports, with minimal long-term data, and no consensus on follow-up [11–14]. Our patient had been in remission for over 3 years on maintenance therapy prior to the transplantation. Given the dual role of MMF in rejection prevention and TAK management [8, 9], anti-TNFɑ was discontinued post-transplant. However, several months after transplantation, MMF had to be withdrawn due to persistent BK virus viremia. Because the patient had maintained long-term remission prior to transplantation and continued steroids and a calcineurin inhibitor, we did not resume anti-TNFɑ treatment but opted for close clinical monitoring. Had there been evidence of Takayasu recurrence and MMF could not be reintroduced, we likely would have restarted infliximab. However, infliximab itself has been associated with an increased risk of BK virus viremia, as demonstrated in the CTOT-19 trial [18], which would have further complicated the clinical decision-making. Given pediatric imaging limitations (radiation and sedation needs), we prioritized clinical monitoring with USD, reserving CTA, PET-CT, or MRA for suspected recurrence. No TAK recurrence occurred during our follow-up.

A summary of key management considerations is provided in Table 1.

**Table 1:** Management recommendations and grading

#	Clinical management point	Recommendation	Grade of evidence
1	**Immunosuppressive induction for infantile TAK**	Initiate treatment with high-dose glucocorticoids and a steroid-sparing agent (e.g., anti-TNFα) based on disease severity and side effect profile	B (Adult and pediatric rheumatology guidelines)
2	**Choice of biologic agent in infants**	Anti-TNFα agents (e.g., infliximab) can be considered for induction and maintenance in infantile TAK, given tolerability and reported effectiveness in small pediatric cohorts	C (Case reports and small case series)
3	**Dialysis modality**	Peritoneal dialysis is the preferred modality in infants with kidney failure, but early transition to hemodialysis may be required to support major abdominal surgery	B (Pediatric nephrology guidelines for modality; case-based decision in TAK)
4	**Timing and indication for vascular surgery**	Major vascular reconstruction (e.g., aorto-aortic bypass) may be needed after disease quiescence to optimize outcomes, including maintenance of distal perfusion and enabling transplant	C (Case series, expert opinion in large-vessel vasculitis)
5	**Renal artery auto-transplantation**	Renal auto-transplantation may be attempted even in the presence of kidney failure to preserve residual kidney function, but outcomes are uncertain in that setting	D (Single-case experience, not generally established)
6	**Timing of kidney transplantation**	Transplantation should be considered after sustained disease remission confirmed by clinical and imaging criteria and after careful consideration of vascular patency	C (Adult and limited pediatric case reports; expert consensus)
7	**Vascular anastomosis strategy**	Anastomosing to iliac vessels may be feasible and safe, even after bypass grafting; preoperative vascular imaging is essential to assess flow and diameter	C (Expert opinion, extrapolated from adult case reports)
8	**Thromboprophylaxis post-transplant**	In patients with extensive vascular reconstruction, consider long-term dual therapy (antiplatelet + anticoagulation) post-transplant, especially after thrombotic events	D (No guidelines; based on single-case outcome and extrapolated reasoning)
9	**Transplantation induction and maintenance after infantile TAK**	In patients with TAK in sustained remission, it may be appropriate to withhold disease-specific biologic therapy post-transplant, provided standard immunosuppression (steroids, calcineurin inhibitor, MMF) is maintained	D (No guidelines; based on single-case outcome and extrapolated reasoning)
10	**Disease recurrence monitoring**	Doppler Ultrasound can be prioritized for routine follow-up in pediatric TAK post-transplant; reserve CTA, PET-CT, or MRA for suspected recurrence or infrequent monitoring	C (Expert opinion and experience; no pediatric-specific imaging guidelines in post-transplant TAK)

Grading key:

• A: High-quality evidence from randomized trials or meta-analyses (rare in pediatric TAK)

• B: Moderate-quality evidence from observational studies or guidelines

• C: Case series, expert consensus, or extrapolated from adult data

• D: Single-case reports or expert opinion in the absence of supportive literature

## Conclusion

This case highlights key management considerations in infantile Takayasu arteritis (TAK) complicated by kidney failure, including the timing of vascular surgery, the role of immunosuppressive therapy, and the feasibility of kidney transplantation after extensive vascular reconstruction. Successful outcome was achieved through coordinated multidisciplinary care involving nephrology, rheumatology, hematology, and surgery. This case supports a stepwise, individualized approach to complex TAK presentations in infancy and underscores the importance of long-term planning for kidney replacement and transplantation. Further research is needed to guide immunosuppression strategies and monitor recurrence risk in this rare population.

## Supplementary Information

Below is the link to the electronic supplementary material.

Graphical abstract (PPTX 420 KB)

## Data Availability

All data generated or analyzed during this study are included in this published article.

## Abbreviations

CKD: Chronic kidney disease
TAK: Takayasu arteritis
USD: Doppler ultrasound
