# Supplementary material for: Navigating complex clinical decisions: kidney transplantation following abdominal aorto-aortic bypass in infantile Takayasu arteritis
Source: Pediatr Nephrol. 2025 Sep 11;41(3):873–81. doi: 10.1007/s00467-025-06932-w (PMC12852304; doi:10.1007/s00467-025-06932-w)
Supplement: Supplementary file 1 — Graphical abstract (PPTX 420 KB) [file 467_2025_6932_MOESM1_ESM.pptx]

## Slide 1
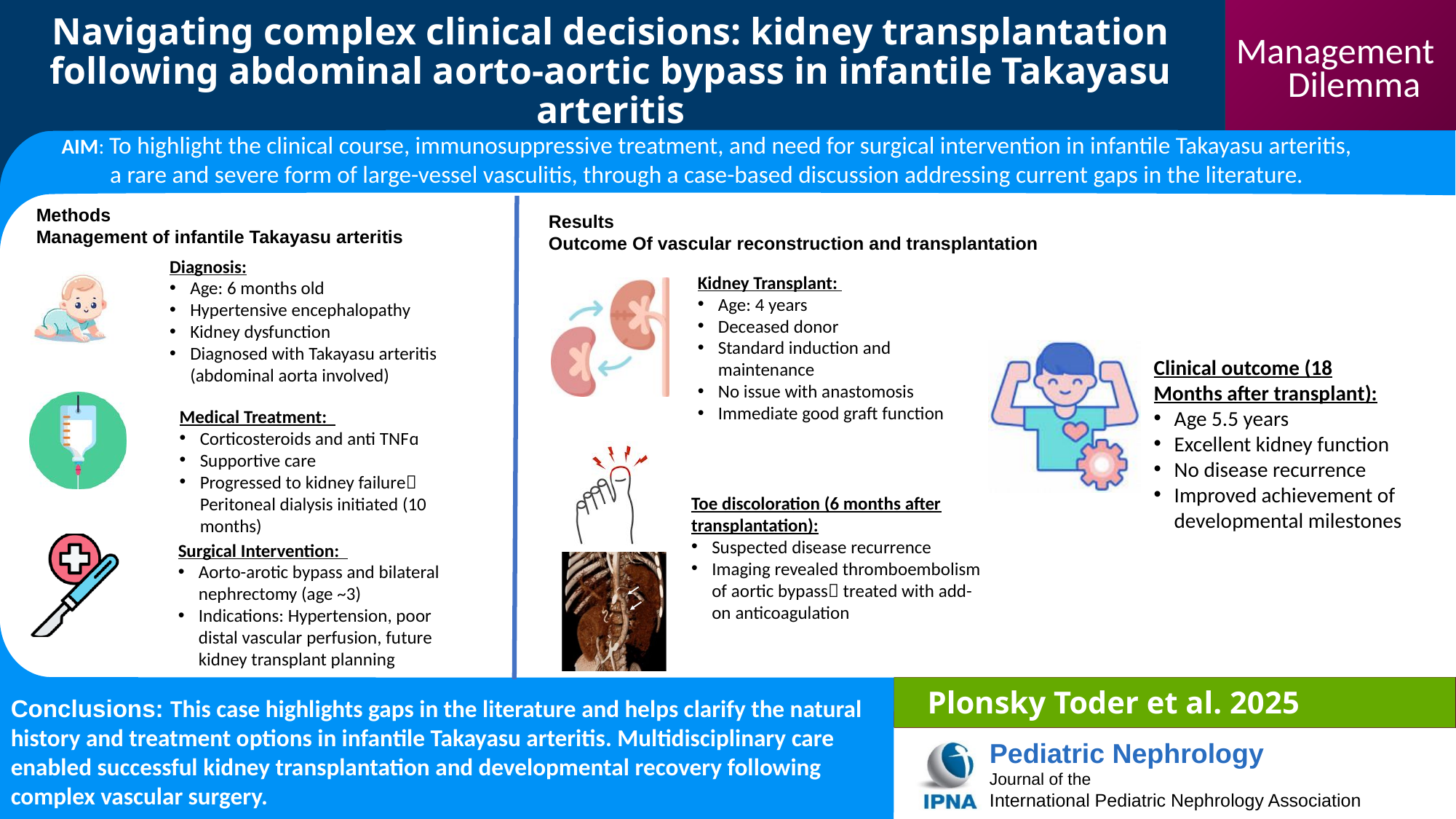

Navigating complex clinical decisions: kidney transplantation following abdominal aorto-aortic bypass in infantile Takayasu arteritis
AIM: To highlight the clinical course, immunosuppressive treatment, and need for surgical intervention in infantile Takayasu arteritis,
a rare and severe form of large-vessel vasculitis, through a case-based discussion addressing current gaps in the literature.
Methods
Management of infantile Takayasu arteritis
Results
Outcome Of vascular reconstruction and transplantation
Diagnosis:
Age: 6 months old
Hypertensive encephalopathy
Kidney dysfunction
Diagnosed with Takayasu arteritis (abdominal aorta involved)
Kidney Transplant:
Age: 4 years
Deceased donor
Standard induction and maintenance
No issue with anastomosis
Immediate good graft function
Clinical outcome (18 Months after transplant):
Age 5.5 years
Excellent kidney function
No disease recurrence
Improved achievement of developmental milestones
Medical Treatment:
Corticosteroids and anti TNFɑ
Supportive care
Progressed to kidney failure Peritoneal dialysis initiated (10 months)
Toe discoloration (6 months after transplantation):
Suspected disease recurrence
Imaging revealed thromboembolism of aortic bypass treated with add-on anticoagulation
Surgical Intervention:
Aorto-arotic bypass and bilateral nephrectomy (age ~3)
Indications: Hypertension, poor distal vascular perfusion, future kidney transplant planning
Plonsky Toder et al. 2025
Conclusions: This case highlights gaps in the literature and helps clarify the natural history and treatment options in infantile Takayasu arteritis. Multidisciplinary care enabled successful kidney transplantation and developmental recovery following complex vascular surgery.
